# Peripheral venous lactate levels substitute arterial lactate levels in the emergency department

**DOI:** 10.1186/s12245-022-00410-y

**Published:** 2022-01-28

**Authors:** Yasufumi Oi, Kosuke Mori, Hidehiro Yamagata, Ayako Nogaki, Tomoaki Takeda, Chikara Watanabe, Yusuke Sakaguchi, Fumihiro Ogawa, Takeru Abe, Shouhei Imaki, Ichiro Takeuchi

**Affiliations:** 1 Emergency Care Department, Yokohama City University Hospital, 3-9 Fukuura, Kanazawa-ku, Yokohama, Kanagawa 236-0004 Japan; 2 Emergency and Critical Care Medical Center, Yokohama Municipal Citizen’s Hospital, 3-9 Fukuura, Kanazawa-ku, Yokohama, Kanagawa 236-0004 Japan; 3 Department of Emergency Medicine, Yokohama City University School of Medicine, 3-9 Fukuura, Kanazawa-ku, Yokohama, Kanagawa 236-0004 Japan; 4 Advanced Critical Care and Emergency Center, Yokohama City University Medical Center, 3-9 Fukuura, Kanazawa-ku, Yokohama, Kanagawa 236-0004 Japan

**Keywords:** Arterial lactate, Peripheral venous lactate, Blood gas analysis, Emergency service, Critical patients

## Abstract

**Background:**

Arterial lactate (AL) level is an important predictor of patient prognosis. AL and peripheral venous lactate (PVL) in blood gas analysis have a low concordance rate, and PVL cannot be used as a substitute for AL. However, if the AL range can be predicted from PVL, PVL may be an alternative method for predicting patient prognosis, and the risk of arterial puncture complications with AL may be reduced. This could be a safe and rapid test method.

**Methods:**

This was a retrospective observational study of 125 cases in which blood gas analysis was performed on both arterial and venous blood with an infectious disease in an emergency department. Spearman’s rank correlation coefficient (*r*) and Bland–Altman analyses were performed. Sensitivity, specificity, and area under the curve (AUC) were calculated for PVL to predict AL < 2 mmol/L or < 4 mmol/L.

**Results:**

The median [interquartile range] AL and PVL were 1.82 [1.25–2.46] vs. 2.08 [1.57–3.28], respectively, r was 0.93 (*p* < 0.0001), and a strong correlation was observed; however, Bland–Altman analysis showed disagreement. When AL < 2 mmol/L was used as the outcome, AUC was 0.970, the PVL cutoff value was 2.55 mmol/L, sensitivity was 85.71%, and specificity was 96.05%. If PVL < 2 mmol/L was the outcome, the sensitivity for AL < 2mmol/L was 100%, and for PVL levels ≥ 3 mmol/L, the specificity was 100%. When AL < 4 mmol/L was used as the outcome, AUC was 0.967, the PVL cutoff value was 3.4 mmol/L, sensitivity was 100%, and specificity was 85.84%. When PVL < 3.5 mmol/L was the outcome, the sensitivity for AL < 4 mmol/L was 100%, and for PVL levels ≥ 4 mmol/L, the specificity was 93.81%.

**Conclusions:**

This study revealed that PVL and AL levels in the same critically ill patients did not perfectly agree with each other but were strongly correlated. Furthermore, the high accuracy for predicting AL ranges from PVL levels explains why PVL levels could be used as a substitute for AL level ranges.

**Supplementary Information:**

The online version contains supplementary material available at 10.1186/s12245-022-00410-y.

## Background

In early emergency care, it is important to promptly determine the severity of a patient’s condition, because severity affects prognosis. Shock, heart failure, severe trauma, and sepsis are the most common pathological conditions that cause lactic acidosis [1]. In patients with these conditions, elevated lactate levels may be associated with morbidity and mortality [2–4]. In patients with shock that could not be differentiated based on the cause, prognosis was poor when lactate levels were higher than 4 mmol/L [5]. In those who survived, lactate levels decreased by 10% within 1 h following treatment initiation [6]. According to these findings, blood lactate levels are useful for evaluating the severity of shock and determining the effects of treatment [7, 8]. Thus, blood gas analyses are performed repeatedly to measure arterial lactate (AL) levels in patients with severe conditions. However, this testing requires arterial puncture and catheterization (arterial line placement) for blood collection, which is invasive and involves a risk of complications [9].

In the emergency department (ED), when determining the effects of treatment, venous blood gas analysis is usually performed as an alternative to arterial blood gas analysis to reduce the risk of complications due to arterial puncture. However, because of disagreement between venous and arterial blood gas analyses, it is necessary to determine the extent to which the values agree between the analyses and whether venous blood gas analysis can substitute for arterial blood gas analysis. Previous studies have reported that parameters in venous blood gas analysis that can substitute for those of arterial blood gas analysis are the hydrogen ion (pH) and bicarbonate ion (HCO3) concentrations. Carbon dioxide partial pressure (pCO_2_), oxygen partial pressure (pO_2_), and lactate levels cannot be used as substitutes [10, 11]. Although pCO_2_ and lactate levels do not match when used as substitutes, parameters in the reference values for venous blood gas analysis provide useful clues for predicting a similar trend to the corresponding values for arterial blood gas analysis [10, 11].

AL is an important parameter for predicting patient prognosis. Septic shock with sepsis-3 is defined as a lactate level ≥ 2 mmol/L with the need for vasopressors to maintain a mean blood pressure of 65 mmHg [12]. Mortality due to septic shock can be estimated using a lactate level ≥ 2 mmol/L instead of lactate clearance [13]. In addition, previous studies have shown that the cutoff lactate level for a poor prognosis is ≥ 3 mmol/L [14, 15] or 4 mmol/L [3, 5, 16, 17]. Thus, despite disagreement between AL and venous lactate (VL) concentrations, VL can be used to predict prognosis in critically ill patients if the AL cutoff can be predicted from VL. According to previous reports that evaluated the relationship between AL and VL levels, when VL levels are within the reference values (< 2 mmol/L), AL levels are also within the reference values (< 2 mmol/L) [18]. Furthermore, when VL levels are ≥ 4.5 mmol/L, AL levels are predicted to be ≥ 4.0 mmol/L [19].

To the best of our knowledge, no studies have confirmed whether VL levels can substitute for ranges of AL levels in critically ill patients. Thus, this study investigated the relationship between VL and AL levels in the same critically ill patients at the time of the initial examination and determined whether VL levels can substitute for ranges of AL levels. If VL levels can be used as a substitute for AL levels, venous blood gas analysis (which reduces the risk of complications associated with arterial puncture required for AL measurement) may be a safer and faster test for critically ill patients.

## Methods

### Study design

This was a retrospective, single center, observational study performed at the Yokohama Municipal Citizen’s Hospital (Yokohama, Japan). Yokohama Municipal Citizen’s Hospital’s catchment area is the central area of Yokohama City, which had an estimated population of 3.7 million in 2020.

### Design

This was a retrospective observational study that examined the relationship between arterial lactate and peripheral venous lactate (PVL) in patients with infection in the emergency department. In the current study, we examined patients who had received arterial and venous blood gas analyses at the time of the initial examination. Venous blood gas analysis was performed to check the condition of patients when we placed the intravenous catheter. Arterial blood gas analysis was performed when blood culture was required or the respiratory status was checked. This study was approved by the Institutional Review Board of Yokohama Municipal Citizen’s Hospital (approval number: 17-07-01). All patients or their families provided informed consent to participate in this study.

### Patients

**Fig. 1:**
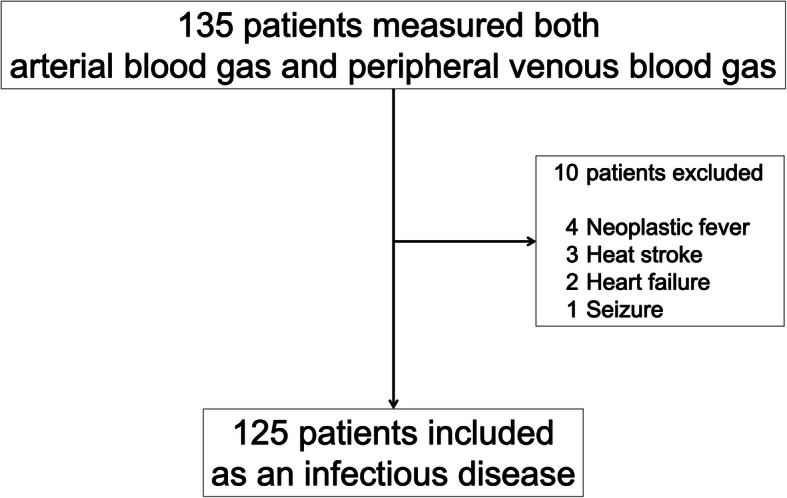
Flow diagram of patients selection

Arterial blood gas analysis and peripheral venous blood gas analysis were performed on the 135 patients in our hospital’s ED from August 2017 to February 2020. When patients were brought to the ED by ambulance, an intravenous line was first established. Then, we collected blood samples and measured venous blood gas. We performed arterial blood gas measurement at the time of the initial examination when the patients needed blood culture or a check of their respiratory condition. In this study, all VL levels were PVL levels. VL and AL were measured only once in the initial examination. One hundred and twenty-five patients had an infection; we excluded 10 patients with other diseases, such as heart failure or heat stroke or neoplastic fever (Fig. 1).

### Blood gas analyzer

Our hospital used the SIEMENS RAPID Point 500 gas analyzer (Siemens Healthcare Diagnostics, Inc., Tarrytown, NY, USA) to measure blood lactate levels. The analyzer can measure values from 0.18 nmol/L to 30 mmol/L.

### Data analysis and statistical methods

Stata 13.1 (Stata Corp., College Station, TX, USA) was used for statistical analyses. Data are presented as medians with interquartile ranges (IQRs) for continuous variables and as numbers and percentages for categorical variables. Student’s *t* test, Mann–Whitney *U* test, Spearman’s correlation, Bland–Altman analysis, and the *χ*^2^ test were used for univariate analysis. Sensitivity, specificity, and area under the curve (AUC) were calculated for PVL to predict AL. Statistical significance was set at *p* < 0.05.

## Results

In the Yokohama Municipal Citizen’s Hospital, we performed arterial blood gas analysis and peripheral venous blood gas analysis in 135 patients from August 2017 to February 2020; 125 patients were diagnosed with infection. The baseline characteristics of the patients are shown in Table 1.

**Table 1:** Characteristics of patients at baseline (*n* = 125)

Characteristic	Frequency (%)* or median (IQR)	
Sex, no.(%)		
Men*	81	(64.8)
Age (years)	81	(72–86)
Systolic blood pressure (mmHg)	134	(116–149)
Heart rate (beats/min)	102	(88–117)
Respiratory rate (breaths/min)	24	(20–29)
Body temperature (°C)	38.5	(37.5–39.2)
SpO_2_ (%)	96	(94–98)
Peripheral venous lactate (mmol/L)	2.08	(1.57–3.28)
Arterial lactate (mmol/L)	1.82	(1.25–2.46)
Arterial-venous puncture time difference (min)	9	(5–17)
Time from arrival ED to blood gas collection (min)	10	(8–13)
Emergency department boarding time (min)	138	(111–181)
Sepsis-3, no.(%)	82	(65.6)
Septic shock, no. (%)	8	(6.40)
SOFA score	2	(1–4)
Death within 28 days, no. (%)	15	(12.0)
Admission, no. (%)	117	(93.4)
Disease type based on ICD-10*, no. (%)		
Certain infectious and parasitic diseases	5	(4.0)
Diseases of the nervous system	1	(0.8)
Diseases of the respiratory system	73	(58.4)
Diseases of the digestive system	20	(16.0)
Diseases of the skin and subcutaneous tissue	6	(4.8)
Diseases of the genitourinary system	18	(14.4)
Injury, poisoning, and certain other consequences of external causes	2	(1.6)

*IQR* interquartile range, *ED* emergency department, *SpO*_*2*_ peripheral oxygen saturation, *ICD* International Classification of Diseases

Regarding baseline characteristics, the most common pathological conditions in the ED were respiratory disorders (73 cases, 58.4%), followed by digestive disorders (20 cases, 16%), and genitourinary disorders (18 cases, 14.4%). The mean age was 81 years (range, 72–86 years). The mean body temperature was 38.5 °C (37.5 °C–39.2 °C), and the mean peripheral oxygen saturation (SpO_2_) was 96% (94–98%). The median AL was 1.82 (1.25–2.46) mmol/L, and PVL was 2.08 (1.57–3.28) mmol/L. Emergency department boarding time was 138 min (111–181 min). The time from arrival at the emergency department to blood gas collection was 10 min (8–13 min). The arterial–peripheral venous puncture time difference was 9 min (5–17 min; all data are expressed in median [IQR]). Eighty-two patients (65.6%) had sepsis-3, and 8 (6.4%) patients had septic shock. Of the patients, 117 (93.6%) were admitted, and 8 (6.4%) received home treatment; 15 (12%) patients died within 28 days of admission, no patient was dead at the time of initial examination.

**Fig. 2:**
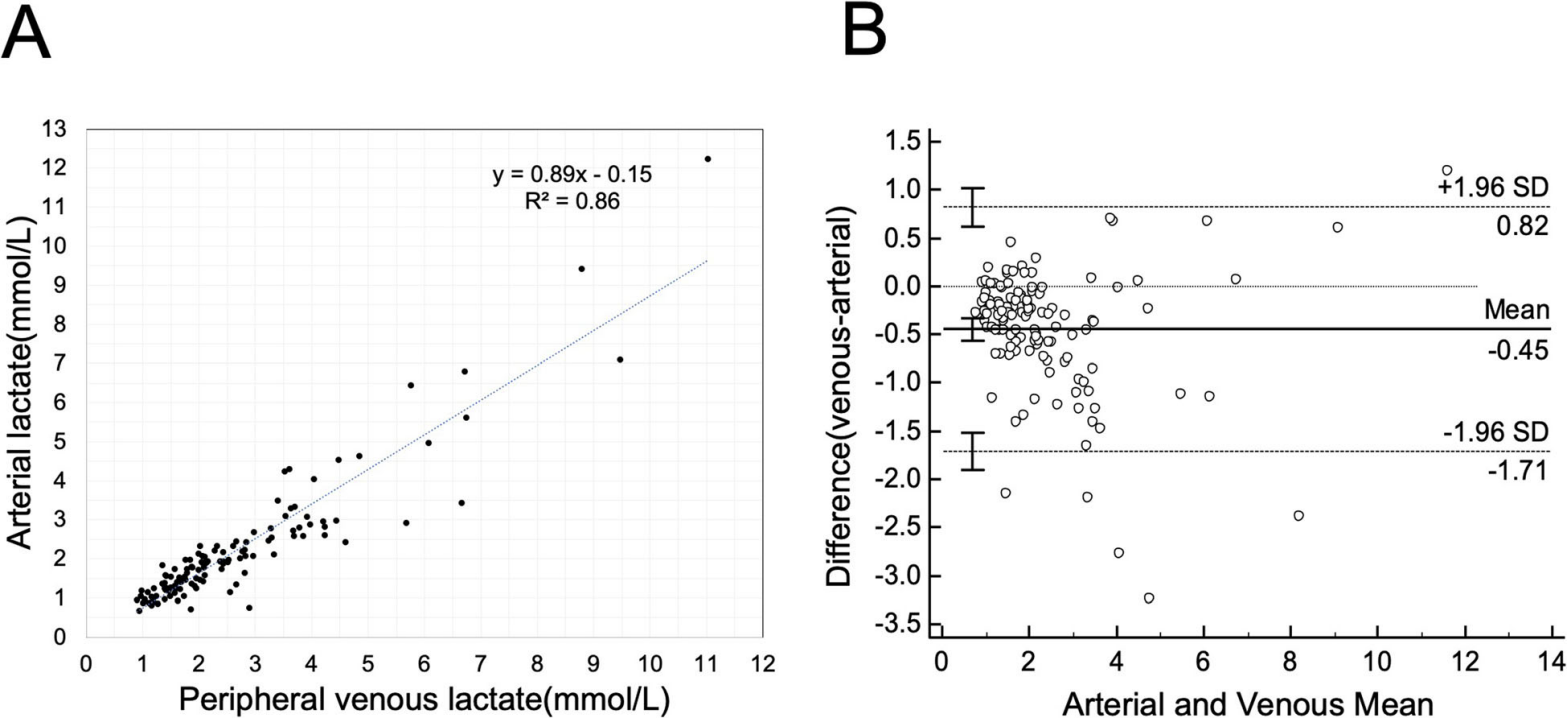
Paired arterial and peripheral venous lactate analysis. **a** Correlation between arterial and peripheral venous lactate levels in individual patients. **b** Bland–Altman bias plot for paired venous and arterial lactate measurements within the same ER. *SD*: standard deviation

The Pearson’s correlation coefficient between AL and PVL was 0.93 (95% CI: 0.90–0.95, *p* < 0.0001; *R*^2^ = 0.86; Fig. 2a). As shown in the Bland–Altman plot (Fig. 2b), the mean difference between AL and PVL was 0.45 ± 0.11 mmol/L. The limits of agreement were between − 1.71 mmol/L and 0.82 mmol/L.

**Fig. 3:**
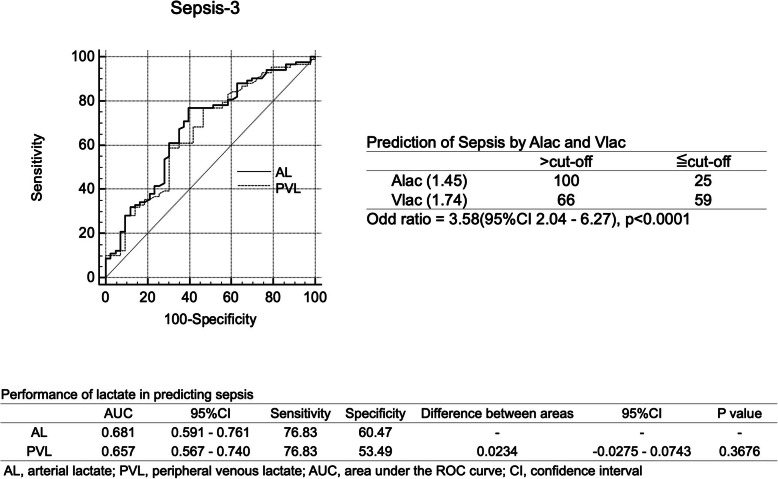
Performance of lactate levels for predicting sepsis. Performance of arterial lactate and peripheral venous lactate in predicting sepsis. *AL*: arterial lactate, *PVL*: peripheral venous lactate, *AUC*: area under the curve, *CI*: confidence interval

**Fig. 4:**
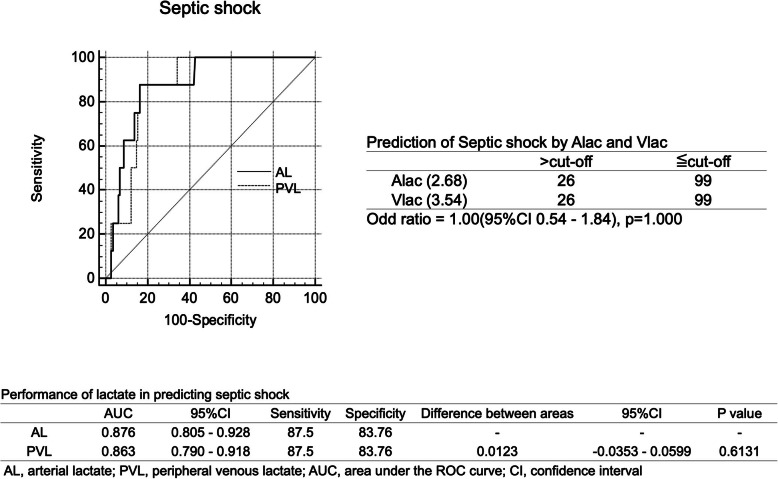
Performance of lactate levels in predicting septic shock. Performance of arterial lactate and peripheral venous lactate in predicting septic shock. *AL*: arterial lactate, *PVL*: peripheral venous lactate, *AUC*: area under the curve, *CI*: confidence interval

AL and PVL levels were comparable predictors of sepsis (AUC: 0.681 vs. 0.657; *p* = 0.368; Fig. 3) and septic shock (AUC: 0.876 vs. 0.863; *p* = 0.613; Fig. 4). The odds ratios (95% CIs) from the cross-tabulation of AL and VL for sepsis and septic shock were 3.58 (2.04–6.27) and 1.00 (0.54–1.84), respectively. Although a partial significant association was observed, there was no significant difference in the accuracy of the AL and PVL levels.

**Fig. 5:**
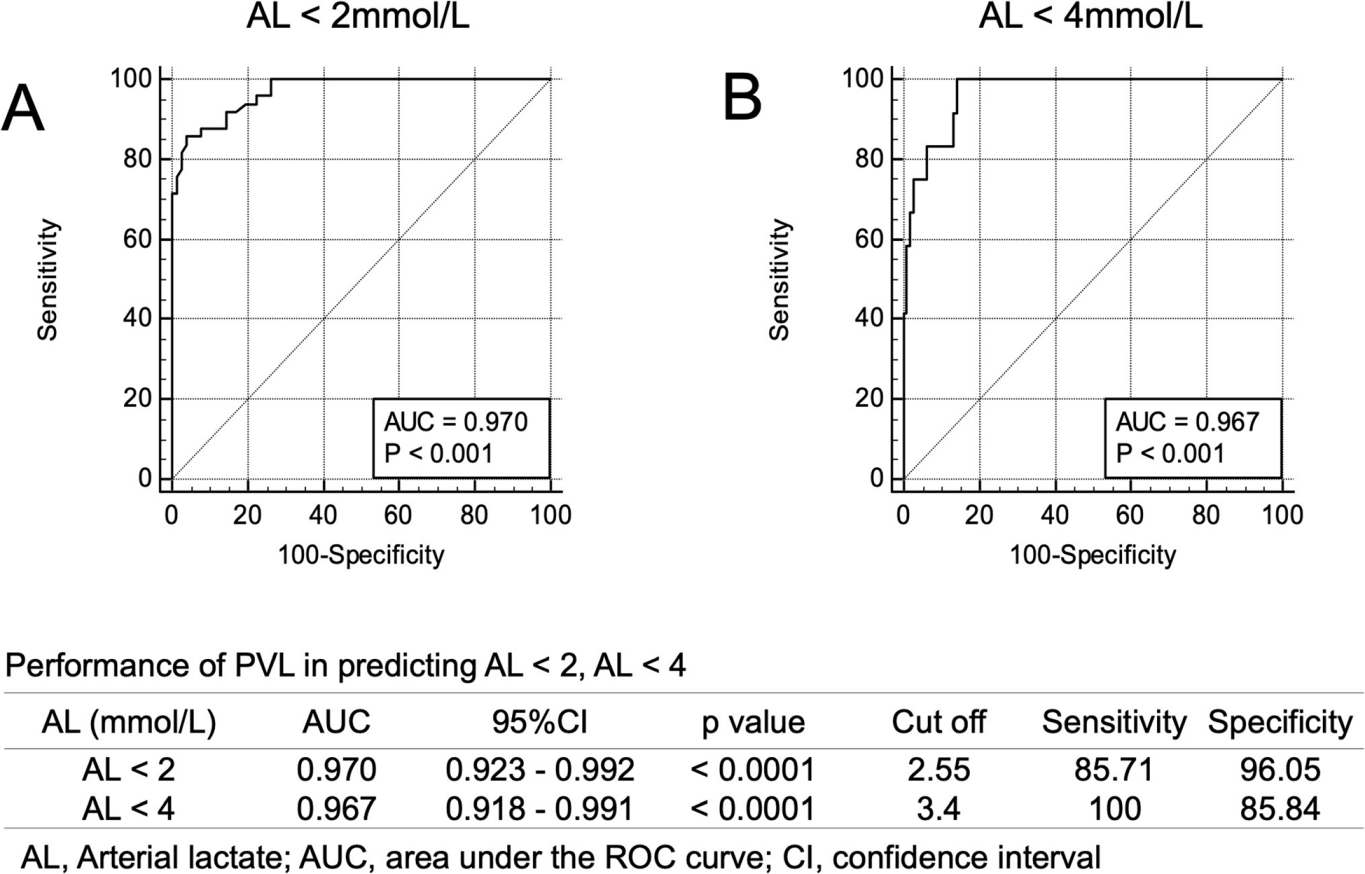
Performance of peripheral venous lactate in predicting arterial lactate levels. **a** Arterial lactate < 2 mmol/L. **b** Arterial lactate < 4 mmol/L. *AL*: arterial lactate, *AUC*: area under the curve, *CI*: confidence interval

**Fig. 6:**
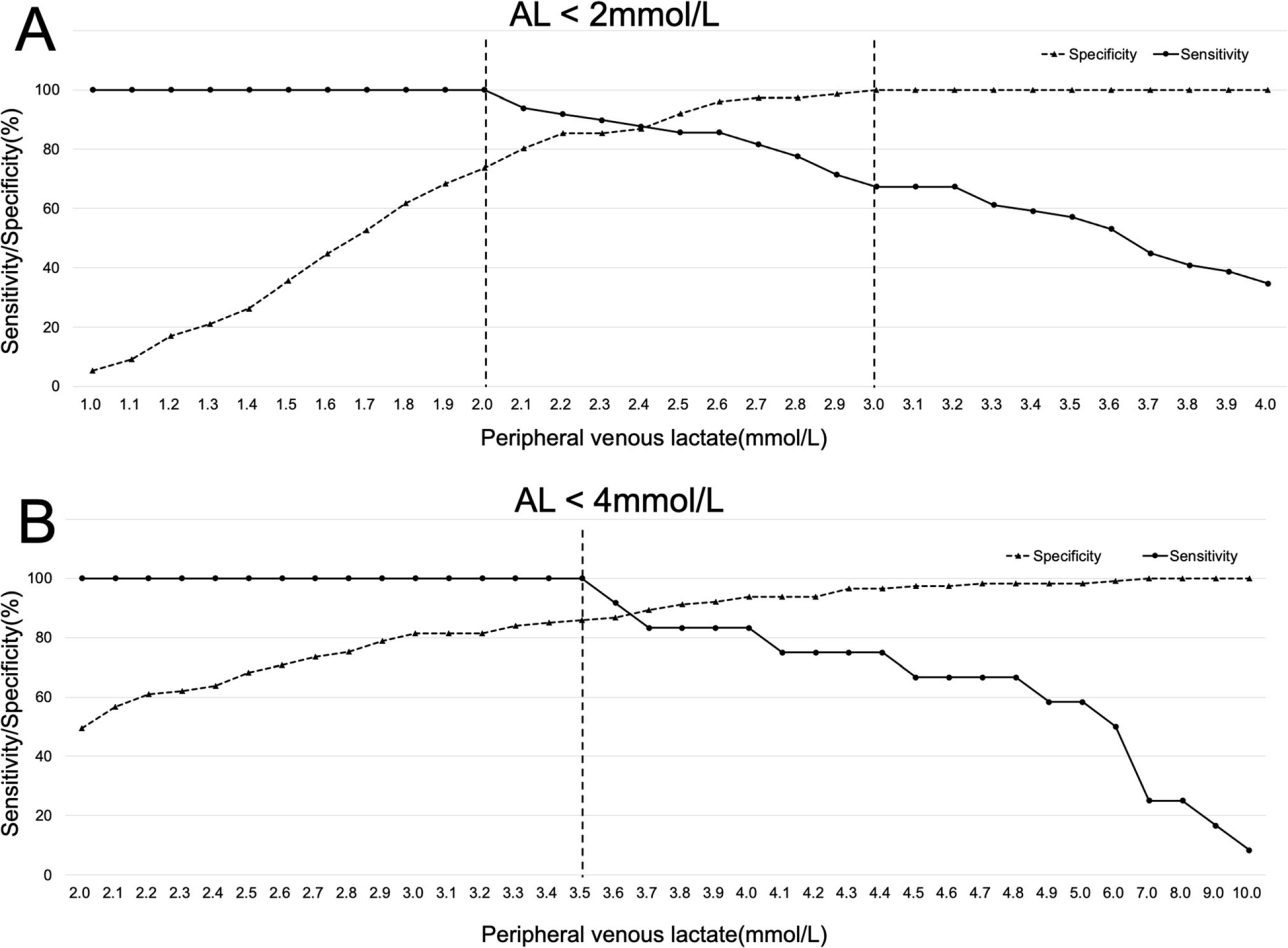
Predicting venous lactate levels lower than arterial lactate levels. **a** Arterial lactate < 2 mmol/L. **b** Arterial lactate < 4 mmol/L

To predict AL levels < 2 mmol/L from PVL levels, the best cutoff value for PVL was 2.55 mmol/L, with a sensitivity and specificity of 85.71 and 96.05, respectively. The area under the receiver operating characteristic (ROC) curve was 0.970 (Fig. 5a). Figure 6a shows the sensitivity and specificity for all PVL levels from which AL levels were predicted to be < 2 mmol/L. When the PVL level was < 2 mmol/L, sensitivity was 100%. In contrast, when PVL levels were ≥ 3 mmol/L, specificity was 100%.

To predict AL levels < 4 mmol/L from PVL levels, the best cutoff value for PVL was 3.4 mmol/L, with a sensitivity and specificity of 100 and 85.84, respectively. The area under the ROC curve was 0.967 (Fig. 5b). Figure 6b shows the sensitivity and specificity of all PVL levels, from which AL levels were predicted to be < 4 mmol/L. When PVL levels were < 3.5 mmol/L, sensitivity was 100%. In comparison, to achieve a specificity of 100%, PVL levels needed to be ≥ 7.0 mmol/L. When PVL levels were ≥ 4.0 mmol/L, as with AL levels, the specificity was 93.81%.

## Discussion

In this study, we investigated the relationship between PVL and AL levels in critically ill patients and determined whether PVL levels could substitute for ranges of AL levels. Our results showed that PVL and AL levels did not perfectly agree, but were strongly correlated. Thus, the high accuracy of predicting ranges of AL levels from PVL levels prompted us to consider PVL levels as a potential substitute for AL levels. In addition, using PVL levels may reduce the risk of complications associated with arterial puncture in critically ill patients.

A previous study showed that VL levels are slightly higher than AL levels; however, VL correlates strongly with AL levels [20]. This finding is consistent with the results in patients with PVL < 3.5 mmol/L in the current study. However, when PVL levels were ≥ 3.5 mmol/L, AL levels were higher than PVL levels in 8 of 28 patients. Another study showed that PVL levels do not agree with AL levels and cannot be substituted for AL levels [21]. We also demonstrated that PVL levels were not a direct substitute for AL levels.

Lactic acidosis is a biomarker of tissue hypoxia caused by an insufficient oxygen supply and indicates poor prognosis [22]. In sepsis, lactate levels are reported to be more strongly associated with mortality than are other parameters [23]. Adverse events occur in patients with sepsis and lactate levels of 2–4 mmol/L [24, 25]. In sepsis-3, septic shock is defined as a lactate level ≥ 2 mmol/L and the need for vasopressors to maintain a mean blood pressure of 65 mmHg [12]. These findings suggest that lactate levels ≥ 2 mmol/L are associated with prognosis.

In another study, patients with shock that could not be differentiated showed poor prognosis when their lactate level was ≥ 4 mmol/L [5]. When patients with infection were examined in three groups (lactate 0–2.5 mmol/L, lactate 2.5–4 mmol/L, and lactate ≥ 4 mmol/L), mortality was 28.4% higher in patients with lactate ≥ 4 mmol/L [3]. Thus, the prognosis of sepsis is poor in patients with lactate levels ≥ 4 mmol/L [16]. Lactate ≥ 4 mmol/L is an indicator of tissue hypoperfusion [17]; thus, this lactate level is considered to be an important cutoff value.

Based on these findings, we conclude that it may be possible to better predict AL levels from venous lactate levels by considering a range of AL levels, rather than a specific value. We examined our results to determine whether AL ranges could be predicted based on PVL levels. First, AL levels were always < 2 mmol/L when PVL levels were < 2 mmol/L, and AL levels were always ≥ 2 mmol/L when PVL levels were ≥ 3 mmol/L, as shown in Fig. 6a. Additionally, we examined patients with AL <4 mmol/L. Figure 6b shows that AL levels were always < 4 mmol/L when PVL levels were ≤ 3.5 mmol/L. Meanwhile, AL levels were always ≥ 4 mmol/L only when PVL levels were ≥ 7 mmol/L; unlike in the 2 mmol/L group, the difference between AL and PVL levels increased. When PVL levels were ≥ 4 mmol/L, 93.8% of our patients had AL levels ≥ 4 mmol/L.

Taken together, our findings revealed that patients with PVL < 2 mmol/L had an AL level < 2 mmol/L, and re-examination of arterial blood gas was unnecessary; this is a strong recommendation for arterial blood gas collection in the emergency department. When PVL levels are 2–3 mmol/L, AL levels are < 4 mmol/L; re-examination is unnecessary to determine whether AL levels are < 4 mmol/L in patients with these PVL levels. Re-examination is only necessary to determine whether AL levels are ≥ 2 mmol/L. When PVL levels are 3–3.5 mmol/L, AL levels are 2–4 mmol/L. Re-examination is unnecessary unless a detailed trend in lactate numerical values needs to be examined. When PVL levels are > 3.5 mmol/L, AL levels are ≥ 2 mmol/L. Re-examination is necessary to determine whether AL levels are ≥ 4 mmol/L and to obtain accurate AL levels for calculating lactate clearance (Table 2).

**Table 2:** Values of AL based on PVL

PVL (mmol/L)	AL (mmol/L)	AL ≧ 2 possibility	AL ≧ 4 possibility	AL check
PVL < 2	AL < 2	No possibility	No possibility	Unnecessary
2 ≦ PVL < 3	0–2 < AL < 4	Potential	No possibility	When necessary to know if AL is 2 mmol/L or more
3 ≦ PVL < 3.5	2 ≦ AL < 4	Potential	No possibility	When necessary to know lactic acid level trend
3.5 ≦ PVL	2–4 ≦ AL	Potential	Potential	When necessary to know if AL is 4 mmol/L or more

*PVL* peripheral venous lactate, *AL* arterial lactate

Thus, among the major vascular complications of femoral artery puncture, pseudoaneurysm, hematoma, arteriovenous fistulas, and retroperitoneal bleeding are mainly caused by technical problems and insufficient bleeding control. It is very important for reducing the risk of complications [26]. In addition, venous blood gas analysis is useful as a clue to know the pathological condition such as whether seizure have recurrence or whether COPD has worsened [27, 28]. If you focus on the lactic acid level, PVL levels are a good marker for predicting the ranges of AL levels. In the ED, venous blood gas analysis appears to be useful for understanding a patient’s condition, and thus reducing the risk of complications related to arterial puncture.

## Limitations

This study has several limitations. First, this was a retrospective, single-center, observational study. Thus, patient selection bias is possible, and our findings lack external validation. Second, variability in technical skill during blood sample collection was not considered, which limits the internal validity of the findings. Third, we did not measure the duration of tourniquet application during venous blood collection, nor did we specify the collection site. Although both venous and arterial blood samples were collected during the initial examination, samples were not collected at the same time, which might have introduced information bias. Bias may be further reduced by standardizing the timing of arterial blood gas collection and the disease and methods of collection. In this study, all venous blood samples were collected from peripheral veins, while most arterial blood samples were collected from the femoral artery. Sonography was not used when puncturing the femoral artery. To minimize limitations in future studies, we suggest collecting the arterial blood gas sample from an A-line secured in the radial artery, and that venous blood gas sample is collected from the upper limbs. This should be done while aiming time difference between samples of 5minutes from each other and also monitoring tourniquet time. Also, we need to evaluate at a younger age group than this study, because patients with sepsis in other countries are younger than this study. Further prospective multicenter studies are required to validate our findings.

## Conclusions

This study revealed that PVL and AL levels in the same critically ill patient did not perfectly agree, but were strongly correlated. Furthermore, the high accuracy of predicting ranges of AL levels from PVL levels explains why PVL levels could be used as a substitute for ranges of AL levels. A prospective multicenter study must be performed to validate our findings.

## Supplementary Information

**Additional file 1.** Title of data: Blood gas analysis data from patients. Description of data: Blood gas analysis data from patients.

## Data Availability

The dataset supporting the conclusions of this article is included within the article (and its Supplementary information files).

## Abbreviations

AL: Arterial lactate
ED: Emergency department
PVL: Peripheral venous lactate
Q-CRT: Quantitative capillary refill time
AUC: Area under the curve
ROC: Receiver operating characteristic curve
pH: Hydrogen ion concentration
HCO3: Bicarbonate
pCO_2_: Carbon dioxide partial pressure
pO_2_: Oxygen partial pressure
IQR: Interquartile range

## References

[1] Kraut JA, Madias NE (2014). Lactic acidosis. N Engl J Med.

[2] Kruse O, Grunnet N, Barfod C (2011). Blood lactate as a predictor for in-hospital mortality in patients admitted acutely to hospital: a systematic review. Scand J Trauma Resusc Emerg Med.

[3] Shapiro NI, Howell MD, Talmor D, Nathanson LA, Lisbon A, Wolfe RE, Weiss JW (2005). Serum lactate as a predictor of mortality in emergency department patients with infection. Ann Emerg Med.

[4] Nichol A, Bailey M, Egi M, Pettila V, French C, Stachowski E, Reade MC, Cooper D, Bellomo R (2011). Dynamic lactate indices as predictors of outcome in critically ill patients. Crit Care.

[5] Broder G, Weil MH (1964). Excess lactate: an index of reversibility of shock in human patients. Science.

[6] Vincent JL, Dufaye P, Berré J, Leeman M, Degaute JP, Kahn RJ (1983). Serial lactate determinations during circulatory shock. Crit Care Med.

[7] Datta D, Walker C, Gray AJ, Graham C (2015). Arterial lactate levels in an emergency department are associated with mortality: a prospective observational cohort study. Emerg Med J.

[8] Bou Chebl R, El Khuri C, Shami A, Rajha E, Faris N, Bachir R (2017). Serum lactate is an independent predictor of hospital mortality in critically ill patients in the emergency department: a retrospective study. Scand J Trauma Resusc Emerg Med.

[9] Scheer B, Perel A, Pfeiffer UJ (2002). Clinical review: complications and risk factors of peripheral arterial catheters used for haemodynamic monitoring in anaesthesia and intensive care medicine. Crit Care.

[10] Bloom BM, Grundlingh J, Bestwick JP, Harris T (2014). The role of venous blood gas in the emergency department: a systematic review and meta-analysis. Eur J Emerg Med.

[11] Byrne AL, Bennett M, Chatterji R, Symons R, Pace NL, Thomas PS (2014). Peripheral venous and arterial blood gas analysis in adults: are they comparable? A systematic review and meta-analysis. Respirology.

[12] Singer M, Deutschman CS, Seymour CW, Shankar-Hari M, Annane D, Bauer M, Bellomo R, Bernard GR, Chiche JD, Coopersmith CM, Hotchkiss RS, Levy MM, Marshall JC, Martin GS, Opal SM, Rubenfeld GD, van der Poll T, Vincent JL, Angus DC (2016). The third international consensus definitions for sepsis and septic shock (Sepsis-3). JAMA.

[13] Ryoo SM, Lee J, Lee YS, Lee JH, Lim KS, Huh JW, Hong SB, Lim CM, Koh Y, Kim WY (2018). Lactate level versus lactate clearance for predicting mortality in patients with septic shock defined by Sepsis-3. Crit Care Med.

[14] Vibert E, Boleslawski E, Cosse C, Adam R, Castaing D, Cherqui D, Naili S, Régimbeau JM, Cunha AS, Truant S, Fleyfel M, Pruvot FR, Paugam-Burtz C, Farges O (2015). Arterial lactate concentration at the end of an elective hepatectomy is an early predictor of the postoperative course and a potential surrogate of intraoperative events. Ann Surg.

[15] Jansen TC, van Bommel J, Schoonderbeek FJ, Sleeswijk Visser SJ, van der Klooster JM, Lima AP, Willemsen SP, Bakker J, LACTATE study group (2010). Early lactate-guided therapy in intensive care unit patients: a multicenter, open-label, randomized controlled trial. Am J Respir Crit Care Med.

[16] Casserly B, Phillips GS, Schorr C, Dellinger RP, Townsend SR, Osborn TM, Reinhart K, Selvakumar N, Levy MM (2015). Lactate measurements in sepsis-induced tissue hypoperfusion: results from the Surviving Sepsis Campaign database. Crit Care Med.

[17] Dellinger RP, Levy MM, Rhodes A, Annane D, Gerlach H, Opal SM (2013). Surviving Sepsis Campaign: international guidelines for management of severe sepsis and septic shock, 2012. Intensive Care Med.

[18] van Tienhoven AJ, van Beers CAJ, Siegert CEH (2019). Agreement between arterial and peripheral venous lactate levels in the ED: a systematic review. Am J Emerg Med.

[19] Theerawit P, Na Petvicharn C, Tangsujaritvijit V, Sutherasan Y (2018). The correlation between arterial lactate and venous lactate in patients with sepsis and septic shock. J Intensive Care Med.

[20] Réminiac F, Saint-Etienne C, Runge I, Ayé DY, Benzekri-Lefevre D, Mathonnet A, Boulain T (2012). Are central venous lactate and arterial lactate interchangeable? A human retrospective study. Anesth Analg.

[21] Paquet AL, Valli V, Philippon AL, Devilliers C, Bloom B, Hausfater P, Riou B, Freund Y (2018). Agreement between arterial and venous lactate in emergency department patients: a prospective study of 157 consecutive patients. Eur J Emerg Med.

[22] Mizock BA, Falk JL (1992). Lactic acidosis in critical illness. Crit Care Med.

[23] Bakker J, Coffernils M, Leon M, Gris P, Vincent JL (1991). Blood lactate levels are superior to oxygen-derived variables in predicting outcome in human septic shock. Chest.

[24] Okorie ON, Dellinger P (2011). Lactate: biomarker and potential therapeutic target. Crit Care Clin.

[25] Tang Y, Choi J, Kim D, Tudtud-Hans L, Li J, Michel A, Baek H, Hurlow A, Wang C, Nguyen HB (2015). Clinical predictors of adverse outcome in severe sepsis patients with lactate 2–4 mM admitted to the hospital. Q J M.

[26] Chen HZ, Liang WS, Yao WF, Liu TX (2021). Compression methods after femoral artery puncture: A protocol for systematic review and network meta-analysis. Medicine.

[27] Turgay YK, Murat Y, Ozge DA, Mustafa S, Ersin A (2014). Can venous blood gas analysis be used for predicting seizure recurrence in emergency department?. World J Emerg Med.

[28] Tricia MM, Glenn H, Gemma H, Catherine R, William K, Tim WH (2016). Using venous blood gas analysis in the assessment of COPD exacerbations: a prospective cohort study. Thorax.

